# Prescribing Patterns and Clinical Effectiveness of Ceftolozane/Tazobactam for ESBL-Producing Enterobacterales: A SPECTRA Real-World Multi-Country Analysis

**DOI:** 10.3390/antibiotics15050423

**Published:** 2026-04-22

**Authors:** Emre Yucel, Alex Soriano, Florian Thalhammer, Stefan Kluge, Mike Allen, Jessica Levy, Huina Yang, Sunny Kaul

**Affiliations:** 1 Merck & Co., Inc., 351 North Sumneytown Pike, P.O. Box 1000, North Wales, PA 19454, USA; 2 Department of Infectious Diseases, Hospital Clinic, Carrer de Villarroel, 170, L’Eixample, 08036 Barcelona, Spain; 3 Department of Urology, Medical University of Vienna, Spitalgasse 23, 1090 Vienna, Austria; 4 University Medical Center Hamburg-Eppendorf, Martini Street 52, 20251 Hamburg, Germany; 5 MSD (UK) Limited, 2 Pancras Sq, London N1C 4AG, UK; 6 Department of Laboratory Medicine, Tan Tock Seng Hospital, 11 Jln Tan Tock Seng, Singapore 308433, Singapore; 7 Royal Brompton & Harefield NHS Foundation Trust, Britten St, London SW3 6PY, UK

**Keywords:** ceftolozane/tazobactam, ESBL-E, real world, clinical outcomes, antimicrobial stewardship, SPECTRA

## Abstract

**Background**: Ceftolozane/tazobactam (C/T) has demonstrated activity against ESBL-producing Enterobacterales (ESBL-E) and provides a carbapenem-sparing option. Broader use of C/T alongside other carbapenem/inhibitor combinations may expand therapeutic choices and reduce selection pressure for carbapenem resistance, supporting antimicrobial stewardship and limiting the spread of carbapenem-resistant Enterobacterales. **Methods**: SPECTRA was a multicenter, retrospective real-world study of hospitalized adults (≥18 years) who received ≥48 h of C/T across seven countries (Australia, Austria, Germany, Italy, Mexico, Spain, and the UK) from January 2016 to November 2020. Medical-record data were collected for up to 6 months before treatment and 30 days after the final C/T dose (or until death). This sub-analysis describes clinical outcomes and healthcare utilization in patients with laboratory-confirmed ESBL-E (n = 39). **Results**: Thirty-nine ESBL-E patients were included (mean age 59.3 years; 56.4% male); 79.5% had ≥1 comorbidity (mean 2.2 per patient). Common pathogens were *Escherichia coli* (n = 23) and *Klebsiella* spp. (n = 12). Investigator-assessed clinical success was 64.9%, microbial eradication was 27.0%, in-hospital mortality was 20.5%, and 30-day readmission was 5.1%. ICU admission during the index hospitalization occurred for 38.5% of patients (mean ICU stay: 16.0 days). The median treatment duration was 11 days while the mean hospital stay after C/T initiation was 13.5 days. **Conclusions**: In this real-world multi-country cohort, C/T showed clinical effectiveness in ESBL-E infections, with outcomes consistent with the overall SPECTRA population. C/T offers a carbapenem-sparing strategy that broadens treatment options and may help reduce reliance on carbapenems, supporting efforts to limit carbapenem-resistant Enterobacterales. The findings warrant evaluation in larger and comparative studies.

## 1. Introduction

Antimicrobial resistance (AMR) is a global public health threat, projected to cause an estimated 10 million deaths per year worldwide and a global economic burden of $100 trillion by 2050 [1,2,3]. Multidrug-resistant (MDR) organisms, such as Gram-negative bacteria *Klebsiella pneumoniae*, *Acinetobacter baumannii*, and *Pseudomonas aeruginosa*, have diminished the efficacy of many traditional antibiotics [3,4,5]. However, the development of new antibiotics has reduced and only a limited number of innovative agents are in development or have been approved in recent years [3,6].

β-lactams in particular remain the single most prescribed antibiotic class [7]. However, due to their widespread use, β-lactamases have developed to hydrolyze the β-lactam amide, providing a mechanism of resistance to these antibiotics [7,8,9]. Class A β-lactamases are the most widely distributed class and include TEM, SHV and CTX-M enzymes [7]. Their efficacy against β-lactams comes from their ability to disseminate themselves onto mobile genetic elements across Gram-negative pathogens, particularly the Enterobacteriaceae family [7]. They also have the unique ability to expand their enzymatic effect to new antibiotics, exhibiting resistance to a wide range of β-lactams, earning them the name extended-spectrum β-lactamases (ESBLs) [7].

Pathogens in the Enterobacterales order that carry ESBL genes, most commonly *Escherichia coli* and *Klebsiella pneumoniae*, are considered one of the most challenging pathogens by the World Health Organization, with an estimated 1.5 billion people colonized worldwide at one time [8]. ESBL-producing Enterobacterales (ESBL-E) have become increasingly prevalent in both healthcare and community settings and can cause pneumonia, bacteremia, and urinary tract infection, among other infections [10]. Their widespread emergence substantially limits therapeutic options and is associated with higher morbidity and mortality among affected patients [8,9,11]

Traditionally, carbapenem is the first choice for treating severe ESBL-E-positive infections in the US and Europe; however, the recent emergence of carbapenemase-producing bacteria has increased the need for innovative therapies that are effective against ESBL-E pathogens [8,12,13]. Innovative therapies such as Ceftolozane/tazobactam (C/T) have emerged to address this, offering potent activity against resistant Gram-negative bacteria including the Enterobacter species [14]. C/T offers patients increased tazobactam exposure at the standard dose when compared to some alternative β lactam/β lactamase inhibitor combinations, which may be relevant when treating isolates with elevated β lactamase expression, such as in ESBL-E infections or in patients with altered drug clearance [15].

While randomized controlled trials (RCTs) remain the gold standard, they often exclude patients with comorbidities, commonly seen in patients with ESBL-E [16,17]. Therefore, real-world evidence is required to compliment findings from RCTs and improve the understanding of treatment effectiveness over a wider range of patient and infection types, and is increasingly used to inform regulatory decisions, clinical guidelines, and health technology assessments [18].

The Study of Prescribing patterns and Effectiveness of Ceftolozane/Tazobactam Real-world Analysis (SPECTRA) provides a large, multi-country dataset of detailed clinical and microbiological data. Previous publications from SPECTRA have summarized its overall findings and disseminated data according to critically ill status in pulmonary disease and respiratory-related infection cohorts [19,20,21]. Understanding current ESBL-E prevalence in patients with severe infections and the effectiveness of alternative treatment options in this population allows for the development of strategies that are effective against these organisms. This analysis focuses specifically on patients with laboratory-confirmed ESBL-E, a prespecified subgroup in the SPECTRA protocol and its approved statistical analysis plan (SAP), and extends prior SPECTRA reports [19,20,21]. Unlike previous SPECTRA publications that described overall prescribing patterns, critically ill patients, or respiratory-related infections, the present manuscript provides ESBL-E-specific clinical outcomes and healthcare resource utilization (HCRU) across multiple countries, addressing an evidence gap relevant to carbapenem-sparing strategies and antimicrobial stewardship.

## 2. Results

### 2.1. Patient Characteristics

A total of 39 of the 617 patients included in the SPECTRA study had ESBL-E-positive infections. Patients with ESBL-E had a mean age of 59.3 years, were 56.4% male and had a mean body mass index (BMI) of 28.6 kg/m^2^ (Table 1). The presence of at least one comorbidity was seen in 79.5% of patients with ESBL-E, with a mean of 2.2 comorbidities per patient (Table 1). Patients could have multiple infections, and the pathogens responsible for the infections were most commonly *Escherichia coli* (n = 23), followed by *Klebsiella species* (n = 12; Supplementary Table S1). Prior antibacterial or antifungal therapy within the last 30 days prior to C/T administration was reported in 28 (71.8%) patients.

### 2.2. Clinical Outcomes

Clinical success was recorded in 64.9% of patients with ESBL-E. When considering the ESBL-E infection type, clinical success was achieved in 59.1% of patients with *Escherichia coli* and 63.6% of patients with *Klebsiella* spp. infections (Table 2). Microbial eradication was confirmed in 27.0% of patients with ESBL-E (Table 2). All-cause in-hospital mortality was 20.5% (95% CI: 9.3, 36.5; Table 2).

### 2.3. Healthcare Resource Utilization

The mean (SD) hospital length of stay following C/T administration was 13.5 (22.5) days (Table 3). During hospitalization, 38.5% of patients were admitted to the ICU, with 40.0% of these admissions related to the index infection (Table 3). The mean (SD) ICU length of stay was 16.0 (17.6) days (Table 3). In total, 5.1% of patients were readmitted to hospital within 30 days following discharge for any cause (Table 3).

## 3. Discussion

In SPECTRA, C/T was administered to 39 patients positive for ESBL-E infections. The two most prevalent species of ESBL-E identified in this study were *Escherichia coli* and *Klebsiella* spp., which is broadly aligned with the previous literature which found that ESBL-producing *Escherichia coli* and *Klebsiella pneumoniae* are the most common ESBL-producing species [22]. *Klebsiella* spp. were one of the most frequently recorded species in this study; however, only one patient was reported as having a *Klebsiella pneumoniae* infection. Apart from this, species information was mostly unspecified. This could be due to a lack of consistent recording of species information in records obtained for this study.

The overall proportion of patients with ESBL-E infections in the SPECTA study (6.3%) was low considering the global prevalence of ESBL-E infections and the growing resistance to carbapenems [8]. This proportion likely reflects the broad real-world scope of C/T use across indications, variable local laboratory testing and reporting practices for ESBL, and the retrospective nature of the study. Screening logs were intended to evaluate representativeness, but incomplete screening-log data and changes in local testing conventions over time may have contributed to under-ascertainment of ESBL-E and selection bias.

Clinical success in patients with ESBL-E (64.9%) was comparable to the total study cohort (67.3%). Clinical success was consistent regardless of the pathogen species (*Escherichia coli* 59.1%, *Klebsiella* spp. 63.6%). These findings suggest that C/T was an effective treatment for patients with ESBL-E, regardless of the pathogen species. However, clinical success observed in this study for patients with ESBL-E treated with C/T was lower than has been reported in the previous literature (83.7%) [23]. This may be due to differences in how the studies defined clinical success, which could be considered more stringent in SPECTRA as patients could not receive further antibiotic treatment following C/T to meet the criteria for clinical success [23]. Differences may also have existed in time to treatment, patient demographics, and clinical infection type and severity, which could also have contributed to the observed differences in clinical success [23].

Notably, the rate of microbial eradication (27.0%) was lower than the clinical success rate (64.9%) in patients with ESBL-E. This discrepancy likely reflects the variable post-treatment sampling practices in routine care and incomplete availability of post-treatment cultures. The SAP specified that microbiology collected within 14 days of the index infection should be analyzed, which likely underestimates microbiological eradication in a retrospective cohort where follow-up cultures are not consistently obtained.

All-cause in-hospital mortality for patients with ESBL-E following C/T treatment (20.5%) was similar to that of the total SPECTRA cohort (21.2%). When compared to previous studies, this result lies within reported mortality rates for patients with ESBL-E across all treatments (12% to 41%), but is higher than previously observed mortality rates for patients with ESBL-E infections treated with C/T (9.8%) [23,24]. However, it should be noted that mortality rates recorded in SPECTRA were for all causes and not specific to the index infection, which may have contributed to the higher observed mortality rates [24]. Interestingly, the mortality rate observed in this study was lower than the 28-day mortality rate recorded in the literature for patients treated with Carbapenems (24.2%) [24]. Comparisons across these studies should be interpreted cautiously due to differences in study design, patient case mix, illness severity, outcome definitions, and follow-up windows and we cannot confirm that these results are due to C/T treatment. However, these findings may suggest the potential of C/T as an acceptable treatment alternative for patients with ESBL-E infections.

ICU admissions and mean ICU length of stay were lower for patients with ESBL-E (38.5% and 16.0 days, respectively) compared to the total study cohort (48.3% and 25.7 days, respectively). In contrast, the previous literature has found that patients with ESBL-E infections experience longer lengths of stay than patients with non-ESBL-E infections [25]. The reasons for patients with ESBL-E reporting lower ICU admissions and length of stay in this study are unclear as the characteristics of the total study cohort and of patients with ESBL-E are similar, and so this does not suggest any underlying differences between the two cohorts.

The level of 30-day all-cause readmission was also lower for patients with ESBL-E infections (5.1%) than for the total SPECTRA population (10.2%). Published data on readmission of patients with ESBL-E infections treated with C/T is limited. Results from the SPECTRA study suggest that C/T treatment in patients with ESBL-E is effective; however, there are limited studies to confirm this.

Across all endpoints considered in this study, the results for patients with ESBL-E infections are comparable to those in the overall study cohort in SPECTRA. Typically, the results for the ESBL-E cohort of the SPECTRA study were also more favorable than seen in the literature for this population. The results of this study therefore suggest that C/T may be an effective treatment in patients with ESBL-E infections and could offer an alternative for reducing carbapenem dependence and treating ESBL-E carbapenem-resistant Enterobacterales.

## 4. Materials and Methods

SPECTRA, a multicenter, retrospective study of adult inpatients treated with C/T for ≥48 h provided real-world outcomes on C/T use in Australia, Austria, Germany, Italy, Mexico, Spain and the UK. Data were extracted from medical records across these seven countries from January 2016 to November 2020. Study design, population and core outcomes are defined in previous papers [19,20,21]. Patients were included if they were aged ≥ 18 years, had received C/T for ≥48 h and had received their last C/T dose ≥ 30 days before data collection. Patients were excluded if they were participating in an interventional clinical trial for Gram-negative infection at the time of treatment.

The primary study objective was to characterize real-world C/T treatment patterns, clinical outcomes and HCRU in hospitalized adults receiving treatment for ≥48 h. A key secondary objective as presented in this manuscript was to analyze patient outcomes according to the presence of ESBL-E infection.

The study was conducted in compliance with the International Society for Pharmacoepidemiology Guidelines for Good Pharmacoepidemiology Practices [26], the ethical principles arising from the Declaration of Helsinki [27], the European Union good pharmacovigilance practices [28], European and national laws with respect to data protection [29], and applicable local regulations.

### 4.1. Outcomes

Data were retrospectively collected from medical records covering up to 30 days after treatment or until death. Outcomes included investigator-assessed clinical success of C/T treatment during index infection, microbial eradication, all-cause in-hospital mortality, length of hospital stay following C/T administration, intensive care unit (ICU) admissions, ICU length of stay, and readmission rates. Severity indicators (systemic inflammatory response syndrome, sepsis, septic shock), ICU admission during the index hospitalization, and antibacterial exposure in the 30 days prior to the index date were captured when documented. Standardized severity scores (clinical pulmonary infection score, sequential organ failure assessment) were collected where available but were sparsely recorded and are not reported as primary results.

Clinical success was defined as microbiological eradication, no Gram-negative therapy needed for a minimum of 48 h after administration of C/T (not including discharge antibiotics or de-escalation), no death due to Gram-negative infection, no additional treatment for exacerbation of respiratory infection within 28 days of stopping C/T, resolution of any exacerbations and no need for re-operation for source control. The clinical success definition was prespecified in the protocol and in the SAP and was operationalized in the electronic Case Report Form (eCRF). Site personnel received an eCRF completion guide and study training to promote consistent adjudication. Investigators completed a standardized ‘Disposition/Discharge’ item indicating whether the index infection was considered a clinical success (Yes/No/Unknown) in accordance with the prespecified criteria. Missing responses were excluded from percentage calculations in line with the SAP. Microbiology data were analyzed if collected within 14 days of C/T initiation and microbiological eradication was defined as negative culture at the site of index infection after C/T.

### 4.2. Statistical Analysis

Categorical variables were reported as counts and percentages and continuous data were reported as mean and standard deviation (SD) or median and interquartile range (IQR). Where available, 95% confidence intervals (CIs) for proportions have been reported. No inferential statistics were performed on this dataset and therefore descriptive statistics are reported only.

## 5. Limitations

This analysis is limited by its descriptive nature and lack of statistical analysis. Accordingly, drawing associations between clinical outcomes and the presence of ESBL-E-positive pathogens is not possible. Furthermore, the small population size (n = 39) of patients with ESBL-E infections limits comparability to the literature, where larger populations are reported. Detailed severity scores were inconsistently available and are therefore limited in this analysis. Prior antibacterial exposure was collected where documented but may be incompletely recorded in the retrospective chart review. Further limitations include the retrospective design, the possibility of multiple records per patient and that data were collected across time windows that changed according to indication.

Despite these limitations, the findings of this SPECTRA sub-analysis offer descriptive, real-world insights into the use of C/T for patients with ESBL-E infections, identifying clinical effectiveness in this population and highlighting a need for further research into C/T as an alternative treatment for ESBL-E-positive patients.

## 6. Conclusions

In this study, C/T demonstrated real-world effectiveness among patients with ESBL-E-positive infections. Clinical success, all-cause in-hospital mortality, ICU admission and length of stay, and 30-day all-cause readmission rates for patients with ESBL-E were similar to those of the total population included in SPECTRA and as seen in the previous literature. These findings suggest that C/T may be a suitable option for treating patients with ESBL-E infections and could offer an alternative for reducing carbapenem dependence and treating ESBL-E carbapenem-resistant Enterobacterales. Further research into C/T use in patients with ESBL-E infections is warranted to confirm the results of this study in a larger cohort and support the adoption of C/T for treating patients with ESBL-E infections.

## Figures and Tables

**Table 1:** Patient characteristics in patients with ESBL-E.

	Patients with ESBL-E (n = 39)	Total Study Cohort (n = 617)
Age *		
Mean (SD)	59.3 (16.5)	57.4 (17.3)
Median	60.0	59.0
Q1;Q3	51.0; 70.0	45.0; 71.0
**Male (%)**	22 (56.4)	404 (65.5)
**BMI (kg/m^2^)**		
Mean (SD)	28.6 (10.9)	25.6 (6.7)
Median	25.6	25.0
Q1;Q3	23.4; 29.4	21.6; 28.2
**Previous care setting**		
Home/Community (%)	29 (74.4%)	495 (80.2)
Other hospital (%)	6 (15.4%)	73 (11.8)
Other skilled care facility (%)	4 (10.3%)	35 (5.7)
Unknown (%)	0	14 (2.3)
**Number of comorbidities**		
≥1 (%)	31 (79.5)	510 (82.7)
Mean (SD)	2.2 (1.7)	2.1 (1.6)
Median	2.0	2.0
Q1;Q3	1.0; 3.0	1.0; 3.0

* For patients aged 90 or older the healthcare provider was asked not to enter the exact age and to check a specific box. Therefore, patients aged 90 or older are included in ‘Missing’ and are not taken into account in the calculation of mean, median, etc. BMI: body mass index; ESBL-E: extended-spectrum β-lactamase-producing Enterobacteriaceae; Q: quartile; SD: standard deviation.

**Table 2:** Clinical outcomes in patients with ESBL-E.

	Patients with ESBL-E (n = 39 *)	Total Study Cohort (n = 617)
**Index infection considered as a clinical success by the investigator (%)**	24 (64.9)	415 (67.3)
*Escherichia coli*	13 (59.1)	N/A
*Klebsiella* spp.	7 (63.6)	N/A
**Microbial eradication (%)**	10 (27.0)	116 (18.8)
**All-cause in-hospital mortality (%)**	8 (20.5)	131 (21.2)
95% CI	(9.3, 36.5)	(18.1, 24.7)

* Clinical success and microbial eradication were not reported in two patients in the ESBL-E cohort. These individuals are considered as missing data and therefore excluded from percentage calculations. CI: confidence interval; ESBL-E: extended-spectrum β-lactamase-producing Enterobacteriaceae; spp: species; N/A: not applicable.

**Table 3:** HCRU in patients with ESBL-E.

	Patients with ESBL-E (n = 39)	Total Study Cohort (n = 617)
**Admission to ICU during the index hospitalization (%)**	15 (38.5)	298 (48.3)
ICU admission related to index infection (%)	6 (40.0)	125 (41.9)
**ICU length of stay (days)**		
Mean (SD)	16.0 (17.6)	25.7 (23.9)
Median	11.0	19.0
Q1;Q3	5.0; 16.0	8.0; 37.5
**30-day all-cause readmission (%)**	2 (5.1)	63 (10.2)
**Post-C/T length of stay (days)**		
Mean (SD)	13.5 (22.5)	15.5 (43.7)
Median	6.0	6.0
Q1;Q3	2.0; 20.0	1.0; 25.0

C/T: Ceftolozane/Tazobactam; ESBL-E: extended-spectrum β-lactamase-producing Enterobacteriaceae; ICU: intensive care unit; Q: quartile; SD: standard deviation.

## Data Availability

The full datasets generated and analyzed to inform the conclusions drawn within this manuscript during the current study are available from the corresponding author upon reasonable request.

## Supplementary Materials

The following supporting information can be downloaded at: https://www.mdpi.com/article/10.3390/antibiotics15050423/s1, Table S1. Pathogen type and site of infection for patients with ESBL-E.
