# Supplementary material for: Prescribing Patterns and Clinical Effectiveness of Ceftolozane/Tazobactam for ESBL-Producing Enterobacterales: A SPECTRA Real-World Multi-Country Analysis
Source: Antibiotics (Basel). 2026 Apr 22;15(5):423. doi: 10.3390/antibiotics15050423 (PMC13203666; doi:10.3390/antibiotics15050423)
Supplement: Supplementary file 1 [file antibiotics-15-00423-s001.zip › antibiotics-4142112-supplementary.pdf]

Supplementary material

Table S1. Pathogen type and site of infection for patients with ESBL-E.

	Patients with ESBL-E (n = 39)
**Pathogen type* (%)**	
Escherichia coli	23 (59.0)
Klebsiella spp	12 (30.8)
Klebsiella pneumoniae	1 (2.6)
Enterobacter spp	3 (7.7)
Morganella morganii	1 (2.6)
**Site of infection (%)**	
Blood	13 (33.3)
Urine	15 (38.5)
Respiratory or upper respiratory	4 (10.3)
Skin and wound or tissue	9 (23.1)
Fluid, pleural fluid, or CSF	3 (7.7)
**Any Antibacterials/Fungal therapy received in the 30 days prior to the Index date**	28 (71.8)

*Total pathogen count is greater than the sum of patients with ESBL-E as patients could have more than one infection. CSF: cerebral spinal fluid; ESBL-E: extended-spectrum β-lactamase-producing Enterobacteriaceae; Spp: species.
